# Toll-Like Receptor-Induced Immune Responses During Early Childhood and Their Associations With Clinical Outcomes Following Acute Illness Among Infants in Sub-Saharan Africa

**DOI:** 10.3389/fimmu.2021.748996

**Published:** 2022-02-03

**Authors:** Luke S. Uebelhoer, Agnes Gwela, Bonnie Thiel, Sophie Nalukwago, John Mukisa, Christopher Lwanga, Justine Getonto, Emily Nyatichi, Grace Dena, Alexander Makazi, Shalton Mwaringa, Ezekiel Mupere, James A. Berkley, Christina L. Lancioni

**Affiliations:** ^1^ Department of Pediatrics, Oregon Health & Science University, Portland, OR, United States; ^2^ KEMRI-Wellcome Trust Research Programme, Kilifi, Kenya; ^3^ Tuberculosis Research Unit (TBRU), Case Western Reserve University, Cleveland, OH, United States; ^4^ Uganda-Case Western Reserve University Research Collaboration, Kampala, Uganda; ^5^ Department of Immunology and Molecular Biology, College of Health Sciences, Makerere University, Kampala, Uganda; ^6^ Department of Pediatrics and Child Health, College of Health Sciences, Makerere University, Kampala, Uganda; ^7^ Centre for Tropical Medicine & Global Health, University of Oxford, Oxford, United Kingdom

**Keywords:** sepsis, toll-like receptor, lipopolysaccharide, malnutrition, pediatric, innate immunity, adaptive immunity

## Abstract

Severely ill children in low- and middle-income countries (LMICs) experience high rates of mortality from a broad range of infectious diseases, with the risk of infection-related death compounded by co-existing undernutrition. How undernutrition and acute illness impact immune responses in young children in LMICs remains understudied, and it is unclear what aspects of immunity are compromised in this highly vulnerable population. To address this knowledge gap, we profiled longitudinal whole blood cytokine responses to Toll-like receptor (TLR) ligands among severely ill children (n=63; 2-23 months old) with varied nutritional backgrounds, enrolled in the CHAIN Network cohort from Kampala, Uganda, and Kilifi, Kenya, and compared these responses to similar-aged well children in local communities (n=41). Cytokine responses to ligands for TLR-4 and TLR-7/8, as well as Staphylococcus enterotoxin B (SEB), demonstrated transient impairment in T cell function among acutely ill children, whereas innate cytokine responses were exaggerated during both acute illness and following clinical recovery. Nutritional status was associated with the magnitude of cytokine responses in all stimulated conditions. Among children who died following hospital discharge or required hospital re-admission, exaggerated production of interleukin-7 (IL-7) to all stimulation conditions, as well as leukopenia with reduced lymphocyte and monocyte counts, were observed. Overall, our findings demonstrate exaggerated innate immune responses to pathogen-associated molecules among acutely ill young children that persist during recovery. Heightened innate immune responses to TLR ligands may contribute to chronic systemic inflammation and dysregulated responses to subsequent infectious challenges. Further delineating mechanisms of innate immune dysregulation in this population should be prioritized to identify novel interventions that promote immune homeostasis and improve outcomes.

## Introduction

Sepsis continues to be a significant cause of pediatric morbidity and mortality worldwide. Although pediatric mortality from sepsis has steadily decreased in well-resourced settings, in low- and middle- income countries (LMICs) the resources required to support patients with sepsis are scarce and outcomes remain exceptionally poor (1–7). Coupled with undernutrition, a highly prevalent condition among young children in LMICs, acute infection manifesting initially as diarrhea or pneumonia often progresses to sepsis, and these common infections continue to be primary drivers of death in children less than 5 years old worldwide (8–11).

Children in LMICs who present to hospital with acute illness are often severely stunted and/or wasted, with underlying environmental enteric dysfunction (EED) (12–15). EED is believed to be a major driver of recurrent infections, immune activation, and chronic inflammation during childhood. Changes in small bowel function accompanied by altered mucosal architecture results in malabsorption and an increase in pathogen translocation that predisposes young children to invasive infection and drives immune dysregulation (12–14, 16–21). Young children with EED often become trapped in a vicious cycle of malnutrition, infection, and inflammation that has been associated with compromised linear growth and neurodevelopment, impaired thymic development, intestinal dysbiosis, and nutritionally-acquired immunodeficiency syndrome (22–31).

The immune response to invasive infection is complex and highly variable due to the multitude of pathogens capable of inducing disease, and underlying host conditions such as age and presence of co-morbidities (32, 33). Studies of critically ill adults and children in resourced settings have suggested that specific immune phenotypes and responses are associated with poor outcomes (34). Both pro- and anti-inflammatory responses are involved: the former contributes to clearance of infection and recovery but can also lead to collateral tissue damage and organ failure; the latter limits local and systemic tissue injury by weakening the immune response, but can lead to secondary infections (33, 35, 36). Cells of the innate immune system, such as monocytes, dendritic cells, and natural killer cells, play a pivotal role in initiation of the immune response against pathogens, and in shaping subsequent adaptive immune responses. Pattern recognition receptors (PRR) on the innate cell surface [toll-like receptors (TLRs) and C-type lectin receptors [CLRs)], in the endosome (TLRs), and in the cytoplasm [retinoic acid-inducible gene-I-like receptors (RLRs) and nucleotide-binding oligomerization domain-like receptors [NLRs)] interact with pathogen-associated molecular patterns (PAMPs) to initiate pro-inflammatory immune responses against pathogens (37). However, the resultant tissue damage from this response releases alarmins and damage-associated molecular patterns (DAMPs) that perpetuate the pro-inflammatory response by autocrine action and can lead to organ dysfunction (38). Anti-inflammatory and tissue-repair immune responses are also elicited during both acute and convalescent phases of severe illness (39), and may contribute to secondary infections among patients who survive their initial infection. This post-sepsis response has been described as “immunoparalysis”, or a state of cellular senescence, where immune cells remain locked in a functionally impaired state by either extrinsic (repeated pathogenic insult, global DAMP expression) or intrinsic (altered T cell repertoires, increased inhibitory receptor expression) factors (40). During sepsis-induced immunoparalysis, there is a massive attrition of lymphocytes that leads to the perpetuation of ongoing infectious foci, an increase in secondary infections, reactivation of latent viremias, and re-admission to hospital with poor outcomes (41–45).

For young children in LMICs, there remains a high mortality rate during hospitalization for severe infection, as well as an increased risk of death for several months following hospital discharge for those children who survived the acute phase of illness (46). The Childhood Acute Illness & Nutrition (CHAIN) Network (47–49) is a network of investigators and sites in LMICs dedicated to understanding biologic and social determinants of survival among highly vulnerable young children during and following severe illness. Working with young, undernourished children in LMICs admitted to hospital with severe illness as part of the CHAIN observational cohort study, and who survived through hospital discharge, we characterized TLR-induced whole blood cytokine responses during acute and convalescent phases of illness. Moreover, we sought to understand if underlying nutritional status was associated with altered host immune responses, and if immune profiles could be used to identify those acutely ill children who would go on to experience an event (death or re-admission) following hospital discharge. Interrogation of immune responses among highly vulnerable young children during severe illness is a critical first step to the identification of specific immunologic pathways associated with poor versus thriving outcomes, and in the development of novel interventions to improve pediatric post-hospitalization outcomes in LMICs.

## Materials and Methods

### Participant Recruitment and Ethics Statement

Participants were enrolled in the Childhood Acute Illness & Nutrition (CHAIN) Network from January 2016-December 2019 (47–49). The CHAIN study protocol was reviewed and approved by the Oxford Tropical Research Ethics Committee (OxTREC), the Scientific and Ethics Review Unit (SERU) of the Kenya Medical Research Institute (KEMRI), and the Makerere University College of Health Sciences, School of Biomedical Sciences Research and Ethics Committee, as well as the institutional review boards of all partner sites. Participating CHAIN sites were Kampala, Uganda, and Kilifi, Kenya. Written informed consent was obtained from a parent or guardian for all participating children prior to enrollment.

### Study Design and Participant Demographics

Children requiring hospitalization at the Kampala and Kilifi CHAIN network sites, who survived their inpatient admission and had blood available for immunologic-based investigations, were eligible for inclusion in the present study. Children were admitted to hospital predominately for severe diarrhea, pneumonia, malaria, and/or anemia, and were commonly found to be malnourished. Children presenting with acute trauma or poisoning were ineligible; for a complete list of inclusion and exclusion criteria in the CHAIN Network study, please refer to Diallo et al. (49). Three subsets of children were included in this analysis: hospitalized children who experienced post-discharge events of re-admission or death (n=22); hospitalized children who did not experience a post-discharge event and survived to month 6 study end point (n=41); non-hospitalized age-matched, well children recruited from local neighborhoods (community participants/CP) serving as a reference group for comparison to hospitalized participants (n=41). Participant demographics are provided in **Table 1**. Nutritional status was stratified by mid-upper arm circumference (MUAC), defined by the CHAIN Network as: not wasted, MUAC ≥12.5cm (age ≥6 months) or MUAC ≥12cm (age <6 months); moderate wasting, MUAC 11.5 to <12.5cm (age ≥6months) or MUAC 11 to <12cm (age <6 months); severe wasting or kwashiorkor, MUAC <11.5cm (age ≥6 months) or MUAC <11cm (age <6 months) or bilateral pedal oedema not explained by other medical causes.

**Table 1:** Participant demographics.

	Hospitalization cohorts		Community participants	P-value
	Post-discharge event (n=22)	No post-discharge event (n=41)	(n=41)	
Recruitment (Uganda)	9 (41%)	15 (37%)	15 (37%)	0.90^3^
Age (months; SD)	11.1 (5.3)	12 (4.7)	12.2 (5.1)	0.71^2^
Sex (female)	9 (41%)	19 (46%)	16 (39%)	0.80^3^
Nutritional status^1^				
Severe wasting	12 (55%)	17 (41%)	0	<0.0001^3^
Moderate wasting	3 (14%)	7 (17%)	2 (5%)	0.21^3^
Non-wasted	7 (32%)	17 (41%)	39 (95%)	<0.0001^3^
Mean MUAC^1^ (cm)	11.19	12.52	13.82	<0.0001^2^
HIV-exposed	5 (23%)	5 (12%)	4 (10%)	0.34^3^
HIV-infected	4 (18%)	1 (2%)	0	0.05^4^

^1^Nutritional status as determined by mid-upper arm circumference (MUAC) at hospital admission (hospitalized cohorts) or enrollment (community participants).

^2^Three-way comparisons performed using ANOVA.

^3^Three-way comparisons performed using Chi squared test.

^4^Two-way comparison between children with and without post-discharge events performed using Fisher's Exact test.

### Whole Blood Cytokine Assay

Blood was collected for immunologic assays within 48 hours of hospital admission, the day of hospital discharge, and 6-months following index hospital admission (among survivors). For CP, blood was obtained at study enrollment only. Peripheral blood was drawn and collected in 2 ml sodium-heparin tubes (BD, catalog #367671) using a standardized SOP for venipuncture that included sterile technique and preparation of the skin with 70% alcohol. 200 μl of undiluted whole blood was aliquoted to individual 2 ml polypropylene tubes (Sarstedt, Germany) pre-prepared with the following stimulants diluted at 5x final concentration in 50 μl RPMI-1640: 1) none (resting condition); 2) ultrapure lipopolysaccharide (LPS, TLR-4 ligand, 0.2 μg/ml, *In vivo*gen, catalog #tlrl-smlps); 3) CL075 (thiazoquinoline derivative, TLR-7/8 ligand, 5 μg/ml, *In vivo*gen, catalog #tlrl-c75-5); 4) staphylococcal enterotoxin B (SEB, monovalent T cell mitogen, 1 μg/ml, Toxin Technology, catalog #BT202). Whole blood stimulation was performed in a water bath at 37°C for 12 hours. Stimulation conditions and incubation time were selected based on previous literature (50–57). To collect materials for cytokine analysis, tubes were spun at 2000 rpm (300 rcf) in a tabletop centrifuge, and the supernatant drawn off and stored at -80°C until batch analysis.

### Multiplex Cytokine Analysis

A custom multiplex cytokine bead array was created using the existing EMD Millipore platform. The following panel of 25 cytokines/chemokines were included: G-CSF, GM-CSF, IFN-α2, IFN-γ, IL-10, MCP-3, IL-12p70, IL-15, sCD40L, IL-17A, IL-1RA, IL-1α, IL-9, IL-1β, IL-2, IL-4, IL-6, IL-7, IL-8, IP-10, MCP-1, Mip-1α, Mip-1β, RANTES, TNF-α. Frozen supernatants were gently thawed and diluted 1:5 using RPMI-1640 prior to performing the assay according to manufacturer’s instructions. All conditions were plated in duplicate. The quality-control samples provided by the manufacturer were included on each plate. A minimum of 50 beads captured was set as the cutoff for each analyte; no analytes fell below this minimum during data acquisition. Plates were run on a Luminex 200™ machine and analyzed using xPONENT^®^ software.

### Research Blood Analysis

All participants had complete blood counts with automated differential performed at all time points. Blood was analyzed using a Coulter Ac*T 5diff CP (Cap Pierce) Hematology Analyzer (Beckman Coulter).

### Statistical Approach

Initial analysis compared resting and stimulated whole blood production of individual cytokines/chemokines between sick, hospitalized children versus children from the same communities (community participants/CP). For the stimulation conditions, background correction was performed prior to analysis and all cytokine/chemokine data underwent log2 transformation to achieve a normalized distribution. For the hospitalized cohort, quantitative cytokine/chemokine results from three time points corresponding to acute illness (hospital admission), early convalescence (hospital discharge), and late convalescence (6-months following initial admission), were compared to those obtained from CP at a single time point. To determine if an individual cytokine/chemokine response in the resting or a stimulation condition was predictive of belonging to the hospitalized versus community cohort, a multivariable logistic regression model incorporating age, sex, recruitment site, and MUAC (at relevant time point) as covariates was applied. Odds ratios and 95% confidence intervals are reported.

To determine the relationship between nutritional status and cytokine/chemokine responses among children in the hospitalized cohort, multivariable linear regression models including age, sex, and site as covariates were applied. Here, individual cytokine/chemokine responses from the resting and stimulation conditions were compared to MUAC at each relevant time point. Parameter estimates, referring to the slope of the relationship between MUAC and the individual cytokine/chemokine response, were calculated; negative and positive parameter estimates denote an indirect or direct relationship, respectively.

A composite cytokine signature that was predictive of belonging to the hospitalized versus community cohort for the resting and each stimulation condition was created, and used to assess the impact of nutritional status on each signature’s predictive value for recent hospitalization. Using a stepwise logistic regression model with both forward and backward elimination for each stimulation condition, individual cytokines and chemokines showing an effect (threshold for selection was p<0.2) on the (log) odds of belonging to the hospitalized cohort were combined to find the best predictive immune signature, with age, sex and site included as covariates. The predictive (log) odds of each cytokine signature were then plotted against MUAC.

In addition, a logistic regression model including MUAC, age, sex, and site was developed to evaluate the relationship between stimulated cytokine/chemokine responses at hospital discharge and the odds ratio of a child experiencing a post-discharge event. Total white blood cell (WBC) and absolute lymphocyte, monocyte, and neutrophil counts were also compared between hospitalized children with and without post-discharge events in an unadjusted analysis, comparing medians to avoid the influence of outliers in data, using the Mann-Whitney test. To determine whether covariates had any effect on these differences, the data was winsorized at the lower 5^th^ and upper 95^th^ percentiles to exclude outliers, and a linear regression model including MUAC, age, sex and site as additional covariates was employed.

Due to the limited sample size of this pilot study, comparisons where unadjusted p-values were ≤0.05 are reported, as are p-values adjusted for multiple comparisons with false discovery rate (FDR) ≤0.1 using the Benjamini-Hochberg procedure.

## Results

### Participants

A total of 63 hospitalized children (24 Kampala; 39 Kilifi) and 41 CPs (15 Kampala; 26 Kilifi) were included for analysis. The demographic breakdown of these participants, including age, sex, MUAC, nutritional status, and HIV status is listed in **Table 1**.

### Children Hospitalized for Severe Illness Have a Reduced Innate Resting Cytokine Profile as Compared to Children in the Community During Both Acute and Convalescent Phases of Illness

**Figure 1:**
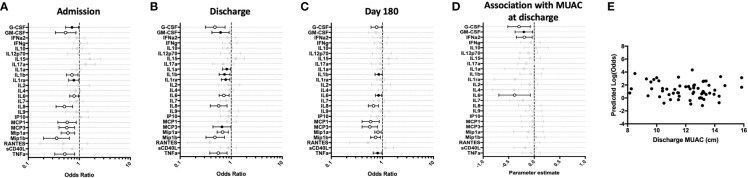
Children hospitalized for severe illness show reduced levels of innate cytokines and chemokines compared to children in the community that persist into convalescence. Whole blood cytokine and chemokine responses were assessed in hospitalized participants at admission to hospital (n=43, **A**), hospital discharge (n=60, **B**), and 180 days-post-discharge (n=51, **C**), and compared to community participants (CP) at enrollment (n=41) using logistic regression taking into account age, sex, recruitment site, and MUAC (at relevant time point). Odds ratios denote higher or lower responses in hospitalized cohort compared to CP cohort. **(D)** Whole blood cytokine and chemokine responses were compared to nutritional status, as measured by MUAC, in hospitalized participants at discharge (n=60) using linear regression taking into account age, sex, and recruitment site. Parameter estimate refers to the slope of the relationship between MUAC and cytokine or chemokine responses; negative and positive parameter estimates denote an indirect or direct relationship, respectively. For **(A–D)**, filled circles indicate significance of p≤0.05; open circles indicate significance of p≤0.1 when adjusting for multiple comparisons. **(E)** To better understand the relationship between cytokine responses and nutritional status in hospitalized participants, a model was created using the cytokine signature (G-CSF, GM-CSF, MCP-3, IL-6, Mip-1-beta) that best discriminated between hospitalized (discharge time point) and CP cohorts. Here, the model adjusted for participant sex, age, and recruitment site, and the predictive strength of the model to reflect recent hospitalization (Y-axis) was plotted against participant MUJAC as measured at hospital discharge (X-axis).

We first examined the resting cytokine profiles in children who were hospitalized for severe illness (hospitalized cohort), as compared to CP. Whole blood cytokine/chemokine responses of hospitalized children at admission, discharge, and at 6-months post-discharge, were compared to CP at a single time point (enrollment). Hospitalized children had lower innate pro-inflammatory cytokine/chemokine levels compared to CP, that persisted throughout the acute and convalescent phases of illness. Lower resting values were predictive of recent hospitalization for numerous innate cytokines (**Figures 1A–C**). No evidence of increased baseline production of pro-inflammatory cytokines was observed in hospitalized children at any time point.

We next examined the relationship between nutritional status and resting immune profiles among hospitalized children during the early convalescent phase of illness when acute illness has resolved (hospital discharge), considering each cytokine individually. Comparing the relationship between MUAC and resting cytokine response, we observed a significant indirect relationship between MUAC and resting values for G-CSF, GM-CSF, IFN-α2, and IL-6 (**Figure 1D**).

To visualize how nutritional status interacts with the resting host cytokine signature during the early convalescent phase of illness, we created a model incorporating the resting whole blood cytokine responses that best discriminate between hospitalized and CP children (G-CSF, GM-CSF, MCP-3, IL-6, Mip-1β), and compared this to nutritional status as indicated by MUAC at time of hospital discharge in only hospitalized children. Here we observed that the identified resting cytokine signature was a poor predictor of recent hospitalization regardless of MUAC (**Figure 1E**).

### Impaired Adaptive Th-1 Responses and Elevated Innate Responses to LPS Are Observed Among Hospitalized Children During the Acute and Convalescent Phases of Illness

**Figure 2:**
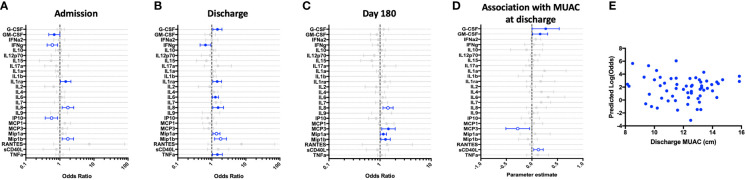
Children hospitalized for severe illness show impaired Th-1 responses and increased innate responses to LPS when compared to children in the community that persist into convalescence. LPS-stimulated whole blood cytokine and chemokine responses were assessed in hospitalized participants at admission to hospital (n=43, **A**), hospital discharge (n=60, **B**), and 180 days-post-discharge (n=51, **C**), and compared to community participants (CP) at enrollment (n=41) using logistic regression taking into account age, sex, recruitment site, and MUAC (at relevant time point). Odds ratios denote higher or lower responses in hospitalized cohort compared to CP cohort. **(D)** Whole blood cytokine and chemokine responses were compared to nutritional status, as measured by MUAC, in hospitalized participants at discharge (n=60) using linear regression taking into account age, sex, and recruitment site. Parameter estimate refers to the slope of the relationship between MUAC and cytokine or chemokine responses; negative and positive parameter estimates denote an indirect or direct relationship, respectively. For **(A–D)**, filled circles indicate significance of p≤0.05; open circles indicate significance of p≤0.1 when adjusting for multiple comparisons. **(E)** To better understand the relationship between cytokine responses and nutritional status in hospitalized participants, a model was created using the cytokine signature (G-CSF, IFNg, Mip-1-beta, IL-6, IL-8) that best discriminated between hospitalized (discharge time point) and CP cohorts. Here, the model adjusted for participant sex, age, and recruitment site, and the predictive strength of the model to reflect recent hospitalization (Y-axis) was plotted against participant MUAC as measured at hospital discharge (X-axis).

To determine if cytokine responses to the TLR-4 agonist LPS were intact among young hospitalized children, we compared LPS-stimulated whole blood responses of the hospitalized cohort to those identified in the CP cohort. A cross-sectional comparison of these responses at hospital admission, discharge, and after 6-months of follow-up demonstrated that, as compared to children in the community, hospitalized children displayed a significantly enhanced innate pro-inflammatory cytokine response to TLR-4 stimulation during acute and early convalescent phases of illness. Increased cytokine values were predictive of recent hospitalization for several cytokines (**Figure 2**). IL-8 and Mip-1β remained elevated at the 6-month follow-up visit (**Figure 2C**). Conversely, hospitalized children showed an impaired Th-1 response to LPS during acute illness, as indicated by decreased IFN-γ and IP-10 responses compared to CP, that resolved during early convalescence. Here, reduced IFN-γ and IP-10 were predictive of recent hospitalization (**Figures 2A–C**).

We next examined the relationship between nutritional status and LPS-induced immune profile among hospitalized children during the early convalescent phase of illness when acute illness has resolved (hospital discharge), looking at each cytokine individually. Comparing the relationship between MUAC and LPS-induced cytokine response, we observed a direct relationship between LPS-induced G-CSF, GM-CSF, and sCD40L, whereas the relationship between MUAC and LPS-induced MCP-3 was indirect (**Figure 2D**).

To visualize how nutritional status interacts with the LPS-induced host cytokine signature during the early convalescent phase of illness, a model was created using the whole blood cytokine signature best discriminating between hospitalized and community children (G-CSF, IFN-γ, Mip-1β, IL-6, IL-8), and compared to nutritional status as indicated by MUAC at time of hospital discharge in only hospitalized children. Here we observed that the identified LPS-induced cytokine signature was highly variable in its ability to predict recent hospitalization regardless of MUAC (**Figure 2E**).

### Innate and Th-1-Specific Adaptive Cytokine Responses to Thiazoquinoline, a TLR-7 and -8 Agonist, Are Impaired During the Acute and Convalescent Phases of Illness

**Figure 3:**
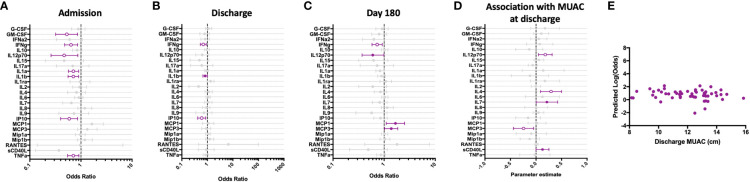
Children hospitalized for severe illness show impaired Th-1 and innate responses to TLR-7/8 stimulation when compared to children in the community that persist into convalescence. CL075-stimulated whole blood cytokine and chemokine responses were assessed in hospitalized participants at admission to hospital (n=43, **A**), hospital discharge (n=60, **B**), and 180 days-post-discharge (n=51, **C**), and compared to community participants (CP) at enrollment (n=41) using logistic regression taking into account age, sex, recruitment site, and MUAC (at relevant time point). Odds ratios denote higher or lower responses in hospitalized cohort compared to CP cohort. **(D)** Whole blood cytokine and chemokine responses were compared to nutritional status, as measured by MUAC, in hospitalized participants at discharge (n=60) using linear regression taking into account age, sex, and recruitment site. Parameter estimate refers to the slope of the relationship between MUAC and cytokine or chemokine responses; negative and positive parameter estimates denote an indirect or direct relationship, respectively. For **(A–D)**, filled circles indicate significance of p≤0.05; open circles indicate significance of p≤0.1 when adjusting for multiple comparisons. **(E)** To better understand the relationship between cytokine responses and nutritional status in hospitalized participants, a model was created using IFNg, a cytokine that best discriminated between hospitalized (discharge time point) and CP cohorts. Here, the model adjusted for participant sex, age, and recruitment site, and the predictive strength of the model to reflect recent hospitalization (Y-axis) was plotted against participant MUAC as measured at hospital discharge (X-axis).

Next, we assessed the immune responses of hospitalized children to thiazoquinoline (CL075), a compound that signals through the TLR-7 and -8 pathways. Whole blood cytokine responses in the hospitalized cohort demonstrated impaired innate and adaptive (Th-1) responses during both acute and convalescent phases of illness, as compared to CP cohort. GM-CSF, IFN-γ, IL-12p70, IL-1α, IL-1β, IP-10, and TNF-α were all lower at hospital admission compared to CP, with depressed IFN-γ and IL-12p70 persisting through 6-months of follow-up. These depressed cytokine values were associated with increased odds of recent hospitalization (**Figures 3A–C**).

We examined the relationship between nutritional status and CL075-induced immune profile among hospitalized children during the early convalescent phase of illness when acute illness has resolved (hospital discharge), looking at each cytokine individually. Comparing the relationship between MUAC and CL075-induced cytokine response, we observed a direct relationship between CL075-induced IL-12p70, IL-4, IL-7, and sCD40L, whereas the relationship between MUAC and CL075-induced MCP-3 was indirect (**Figure 3D**).

We next visualized how nutritional status interacts with CL075-induced host cytokine signature during the early convalescent phase of illness. A model was created using the whole blood cytokine signature best discriminating between hospitalized and community children (IFN-γ only), and compared to MUAC at hospital discharge among hospitalized children. We observed no clear relationship between the predictive value of CL075-induced IFN-γ responses for recent hospitalization and nutritional status as reflected by MUAC (**Figure 3E**).

### Staphylococcus Enterotoxin B-Induced T Cell Responses in Whole Blood of Hospitalized Children Are Impaired During Acute Illness but Improve During Convalescence

**Figure 4:**
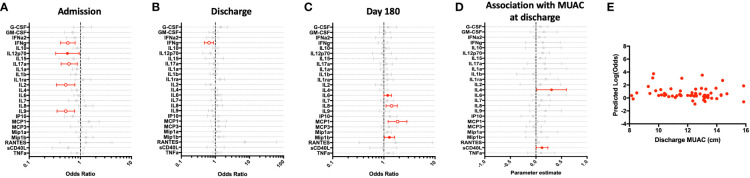
Children hospitalized for severe illness exhibit an impaired T cell response to polyclonal stimulation when compared to children in the community that improves during convalescence. SEB-stimulated whole blood cytokine and chemokine responses were assessed in hospitalized participants at admission to hospital (n=43, **A**), hospital discharge (n=60, **B**), and 180 days-post-discharge (n=51, **C**), and compared to community participants (CP) at enrollment (n=41) using logistic regression taking into account age, sex, recruitment site, and MUAC. Odds ratios denote higher or lower responses in hospitalized cohort compared to CP cohort. **(D)** Whole blood cytokine and chemokine responses were compared to nutritional status, as measured by MUAC, in hospitalized participants at discharge (n=60) using linear regression taking into account age, sex, and recruitment site. Parameter estimate refers to the slope of the relationship between MUAC and cytokine or chemokine responses; negative and positive parameter estimates denote an indirect or direct relationship, respectively. For **(A–D)**, filled circles indicate significance of p≤0.05; open circles indicate significance of p≤0.1 when adjusting for multiple comparisons. **(E)** To better understand the relationship between cytokine responses and nutritional status in hospitalized participants, a model was created using IFNg, a cytokine that best discriminated between hospitalized (discharge time point) and CP cohorts. Here, the model adjusted for participant sex, age, and recruitment site, and the predictive strength of the model to reflect recent hospitalization (Y-axis) was plotted against participant MUAC as measured at hospital discharge (X-axis).

To assess T cell responses to polyclonal activation, we stimulated whole blood with the superantigen staphylococcus enterotoxin B (SEB). As compared to children in the community, T cell responses to SEB were transiently impaired at hospital admission, as indicated by decreased IFN-γ, IL-2, IL-9, and IL-17A (**Figure 4A**). However, all T cell responses, except for IFN-γ, had recovered to that seen in CP by hospital discharge (**Figure 4B**). Further, when assessed at 6-month follow-up, these responses were equivalent to those observed in CP (**Figure 4C**). Notably, innate cytokine responses (IL-6, IL-8, MCP-1 and Mip-1β) were elevated during the last convalescence phase of illness as compared to children in the community (**Figure 4C**).

We examined the relationship between nutritional status and SEB-induced immune profile among hospitalized children during the early convalescent phase of illness when acute illness has resolved (hospital discharge), looking at each cytokine individually. Comparing the relationship between MUAC and SEB-induced cytokine response, we observed a direct relationship between SEB-induced IL-4 and sCD40L; no other direct or indirect relationships were observed (**Figure 4D**).

We next visualized how nutritional status interacts with SEB-induced host cytokine signature during the early convalescent phase of illness. A model was created using the whole blood cytokine signature best discriminating between hospitalized and community children (IFN-γ only), and compared to MUAC at hospital discharge among hospitalized children. Here we observed no clear relationship between the predictive value of SEB-induced IFN-γ responses for recent hospitalization and nutritional status as reflected by MUAC (**Figure 4E**).

### White Blood Cell Counts and IL-7 Production Are Altered Among Children With Post-Discharge Events

**Figure 5:**
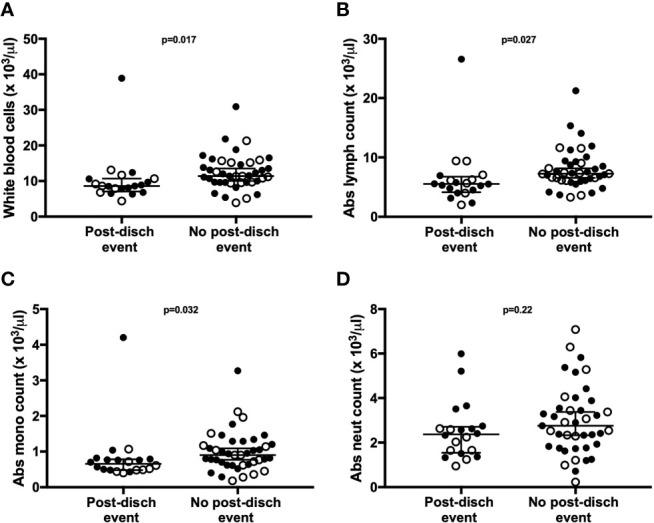
Total lymphocyte, leukocyte, and monocyte counts are associated with post-discharge adverse events in children hospitalized for severe illness. Complete blood counts with differential were performed at hospital discharge and compared between participants with (n=20) and without (n=41) post-discharge events of death or re-admission to hospital. Shown are median values with interquartile range for white blood cell counts **(A)**, absolute lymphocyte counts **(B)**, absolute monocyte counts **(C)**, and absolute neutrophil counts **(D)**, displayed as cell number x10 ^3^/μl of blood. P-values were calculated using Mann-Whitney test. Filled circles and open circles indicate participants from Kilifi, Kenya and Kampala, Uganda sites, respectively.

**Figure 6:**
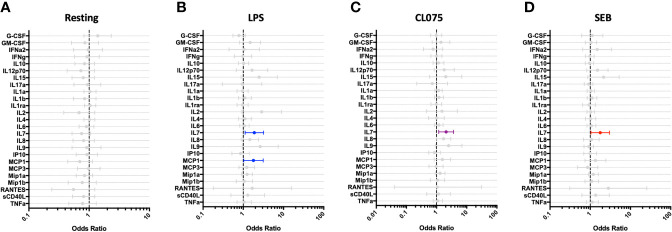
IL-7 responses to multiple stimuli are altered among hospitalized children who experience post-discharge adverse events. Whole blood cytokine and chemokine responses at hospital discharge were compared between participants who experienced a post-hospital-discharge event of death or re-admission (n=22) and those who completed 6-months of follow-up with no event (n=41). Shown are odds ratios of experiencing post-discharge event in unstimulated **(A)** and PAMP-stimulated **(B–D)** whole blood, using logistic regression taking into account age, sex, recruitment site, and MUAC. Filled circles indicate significance of p≤0.05; open circles indicate significance of p≤0.1 when adjusting for multiple comparisons.

Children who experienced post-discharge events of hospital re-admission or death showed significantly lower total white blood cell counts, including absolute lymphocyte and monocyte counts, when compared to children who did not experience post-discharge events throughout 6-months of follow-up. This effect remained significant when the data was adjusted using a linear model taking into account MUAC, age, sex and site (**Figure 5**). No difference in absolute neutrophil count was noted in this analysis (**Figure 5D**). In addition, whole blood immune responses to TLRs and SEB were compared between children with and without post-discharge events (**Figure 6**). Increased IL-7 production to all tested stimuli among children with post-discharge events was observed. This increase in IL-7 responses was associated with increased odds of a post-discharge event (**Figures 6B–D**). No significant association between IL-7 levels and absolute lymphocyte counts were found in children with post-discharge events (data not shown). There was no significant impairment in cytokine production to any tested stimuli observed when comparing children with and without post-discharge events (**Figures 6A–D**).

## Discussion

In this study we examined resting and stimulated whole blood cytokine responses among young children living in LMICs, including children who required hospitalization for severe illness and well children recruited from local communities. The TLR agonists utilized in this study served as a method of interrogating functional host immune responses that may have been deranged as a result of severe acute illness and undernutrition, both common among children in LMICs. We specifically selected LPS, an agonist for TLR-4, to model innate responses to gram-negative pathogens that commonly cause severe illness in this population (58). CL075, a dual agonist for TLR-7 and TLR-8 that mimics single-stranded RNA, was selected for study given the high burden of viral infections in young children.

Based on prior studies of severely ill adults and children performed in well-resourced settings (40, 59–62), we anticipated that hospitalized children would exhibit limited pro-inflammatory cytokine/chemokine responses to the tested stimuli, with malnourished children and children who experience post-discharge events exhibiting the most muted responses. Our data revealed that the immune response to each stimulus was distinct, with heightened innate cytokine responses to LPS among hospitalized children that persisted through late convalescence, while cytokine responses to CL075 were compromised during both acute and early convalescent stages of illness. Responses to the T cell mitogen SEB demonstrated compromised production of several T-cell-associated cytokines (IFN-γ, IL-17A, IL-2, and IL-9) during acute illness, with exaggerated production of pro-inflammatory innate cytokines continuing through late convalescence. Our data suggest that the hospitalized cohort studied here may in fact not be suffering innate immune cell deficits; rather, the innate immune compartment in these children is primed for heightened responses to LPS. Children with post-discharge readmission or death were found to have significant depression in total white blood cell counts at the time of discharge, as well as lymphocyte and monocyte counts, a finding that mirrors similar observations made in adult sepsis cohorts from well-resourced settings (34, 40, 63). Surprisingly, there was no evidence of a compromised pro-inflammatory cytokine response at discharge among children with post-discharge events; rather, heightened production of IL-7 was observed against all tested stimuli. Although we did not identify a statistically significant correlation between IL-7 production and absolute lymphocyte counts in our limited sample size, our findings suggest a heightened drive to restore T cell homeostasis among children who would go on to have poor outcomes.

Our comparison of resting (unstimulated) and stimulated innate cytokine profiles among hospitalized children revealed unexpected findings. Specifically, unstimulated samples from hospitalized children exhibited lower levels of numerous innate cytokines during both acute and convalescent phases of illness as compared to children in the community. Interestingly, innate responses to LPS in this same cohort were elevated throughout the length of this study. This contrasts with a previously published study in a well-resourced setting suggesting that children with severe illness experience immunoparalysis and innate immune deficiencies (64). We also found no evidence for “LPS tolerance,” a phenomenon where innate immune cells undergo metabolic and epigenetic alterations due to repeat immune challenge, resulting in cellular reprogramming and an overall reduction in the pro-inflammatory response (65–68). Rather, the sustained, altered innate cytokine responses to TLR-4 stimulation in our current young cohort is suggestive of the phenomenon of trained immunity. Trained immunity has been described following immunization with Bacillus Calmette-Guérin, as well as exposure to PAMPs including LPS (69) and β-glucan. Trained immunity refers to epigenetic reprogramming of innate cells that develops following an infectious insult or vaccination, leading to long term alterations in functional responses to subsequent stimuli (70, 71). We hypothesize that some hospitalized children in this cohort may have experienced an acute infection with a gram-negative pathogen that altered their long-term response to subsequent *in vitro* LPS-stimulations. In support of this, de Laval and colleagues recently demonstrated persistent immune alterations in mice after LPS stimulation that increased responsiveness of associated immune genes to secondary stimulation (69).

CL075 is a dual stimulant of endosomal TLR-7/8, targeting TLR-7-expressing plasmacytoid dendritic cells (pDCs) and B cells, and TLR-8-expressing monocytes and myeloid dendritic cells (mDCs) (72). TLR-7 activation in pDCs classically drives intracellular signaling *via* MyD88, induction of IRF-7, and endpoint secretion of IFN-α for anti-viral defense. Conversely, activation of TLR-8 in mDCs *via* the same signaling cascade results in secretion of IL-12p70, which polarizes activated naïve T cells towards IFN-γ-secreting Th-1 subsets. When compared to community counterparts, hospitalized children exhibited a sustained impairment in the secretion of all 3 cytokines (IFN-α, IL-12p70 & IFN-γ; **Figures 3A–C**), suggesting an upstream impact of illness and malnutrition on DC function. Furthermore, our data showed that IL-12p70 secretion was directly linked to MUAC (**Figure 3D**) even after adjusting for multiple comparisons, indicating that poor nutritional status may directly exacerbate poor DC function. A previous study assessing the impact of malnutrition on DC function in 81 Zambian children reported decreased total numbers and low activation during acute infection, which improved after recovery from illness. However, a subset of children (17%) with endotoxemia had ‘anergic’ type immature IL-10-secreting DCs which failed to drive T cell proliferation (73). DCs are pivotal regulators of immunity, performing individual innate functions and playing a key role in kick-starting adaptive immune responses. Failure of upstream DC responses thus have an obvious negative impact on downstream T cell responses, and we hypothesize that the altered response to LPS, low levels of IFN-γ in hospitalized children, and dysregulated IL-7 secretion could be a consequence of impaired DC responses to TLR-stimulation. Our evidence highlights the DC-T cell activation axis as a novel immune pathway for target therapies aiming to improve both innate and adaptive immunity among young children.

The hospitalized cohort presented here is potentially confounded by differences in nutritional status, with many children experiencing severe wasting in addition to severe, acute illness. Examining cytokine/chemokine responses in our whole blood assay among hospitalized children only (children in CP cohort were predominantly not wasted), we found that higher cytokines responses were directly related to improved nutritional status, as determined by MUAC. The notable exception to this was monocyte-chemotactic protein 3 (MCP-3), which showed an inverse relationship with MUAC. Both MCP-1 and MCP-3 are readily produced by adipocytes which are highly responsive to metabolic status and able to secrete bioactive molecules with downstream effects on immunity (74). Although MCP-1 is known to be increased in obese adults (75), as well as malnourished Ugandan children who have undergone re-feeding therapy (76), MCP-3 was not assessed in these prior studies. To our knowledge, this is the first report of MCP-3 expression in the context of infant nutritional status.

In addition to assessing the relationship between production of individual cytokines and nutritional status, we developed a cytokine signature to each tested stimulus and examined its relationship with MUAC. Here we observed that the resting and LPS-induced cytokine signatures were highly variable in their ability to predict recent acute illness among children, and this was not altered or improved by nutritional status. When only one dominant cytokine was utilized (IFN-γ), as when examining the relationship between CL075- and SEB-induced cytokine response and MUAC, the ability of the cytokine response to predict recent hospitalization was more consistent regardless of nutritional status. Our analysis indicates that composite cytokine and chemokine signature modeling in the context of nutritionally diverse severely ill pediatric cohorts may not be ideal for small sample sizes, as was available in this analysis.

Despite implementation of international guidelines, undernourished young children in LMICs with acute illness continue to have a markedly increased risk of death during hospital admission that persists following discharge. To further define risk factors that contribute to these post-discharge events, we analyzed the immune responses in our hospitalized cohort by separating children who died or were re-admitted to hospital from those who successfully completed 6-months of follow-up. Of all cytokines and chemokines analyzed, only increased IL-7 responses were associated with post-discharge events. IL-7 is a master regulator of both the naïve and memory T cell compartments; signaling through the IL-7 receptor is necessary for death, survival, and turnover of both CD4+ and CD8+ T cells (77). Low levels of IL-7 are detectable in human serum, and increased IL-7 production is thought to be a compensatory effect of lymphopenia in numerous disease states (78–82). Notably, IL-7 replacement therapy was recently studied in a Phase IIb trial among adults with sepsis (83). Although the study was not powered to observe difference in mortality, and baseline IL-7 levels were not reported, patients who received IL-7 therapy had significant increases in CD4+ and CD8+ T cell counts, and the treatment was well tolerated. We reviewed complete blood counts in our hospitalized cohort, and found significant decreases in total white blood cell, lymphocyte, and monocyte counts. It is likely that increased IL-7 production seen in children with post-discharge events reflects an increased drive for T cell proliferation and reduction in T cell apoptosis in an effort to restore adaptive immune homeostasis. Given these children had poor outcomes, this IL-7 compensatory response may have been insufficient. Future studies in larger cohorts of young children are critical to identify specific targets for immune-based therapeutics that improve outcomes from severe infection.

Our study provides a longitudinal assessment of immune responses in an East African cohort of young children, with varied nutritional and illness status. It is important to note that this pilot study had a limited sample size, and children were hospitalized with a diversity of serious illnesses that limited our capacity to interrogate immune function in the context of specific presenting illness. Our study did not include cell phenotyping by flow cytometry, and thus we were unable to assess cellular phenotype and its association with disease state and outcome. However, the longitudinal nature of our study with defined clinical outcomes and access to a reference population of well children from the same community, combined with interrogation of two distinct TLR pathways and the mitogen SEB, has provided comprehensive insight into how serious acute illness in early childhood is associated with perturbations in both acute and long-term immune function and clinical outcomes. Importantly, our data suggest that the immune system of a child hospitalized for severe illness in LMICs is altered during the acute illness phase, as well as the early and convalescent phases of recovery. Notably, heightened innate responses to LPS and reduced Th-1 responses to CL075 persisted for up to 6 months following hospitalization, and may predispose children to a dysregulated immune response to subsequent infectious challenge. We have also shown that children who go on to have poor outcomes following hospitalization exhibit leukopenia at hospital discharge, as previously reported in well-resourced settings, and a trend towards heightened IL-7 response to all tested stimuli. These findings suggest that acute severe illness during early childhood may have a lasting impact on immune function, and emphasizes the importance of performing longitudinal studies to better understand how illness-associated immune dysregulation can be corrected to promote healthy outcomes.

## Data Availability Statement

The raw data supporting the conclusions of this article will be made available by the authors, without undue reservation.

## Ethics Statement

The studies involving human participants were reviewed and approved by the Oxford Tropical Research Ethics Committee (OxTREC), the Scientific and Ethics Review Unit (SERU) of the Kenya Medical Research Institute (KEMRI), and the Makerere University College of Health Sciences, School of Biomedical Sciences Research and Ethics Committee. Written informed consent to participate in this study was provided by the participants’ legal guardian/next of kin.

## Author Contributions

LU and AG contributed equally to the study. LU, AG, JB, and CLL contributed to conception and design of the study. LU, AG, SN, JG, EN, JM, CL, GD, AM, SM, and EM contributed to assay development and validation, performed and/or processed material for experiments, or were directly involved with participant enrollment and/or collection of essential materials or data. BT performed all statistical analyses. LU, CLL, AG, and JB contributed to writing the manuscript. All authors reviewed the manuscript and approved the submitted version.

## Funding

This work was supported, in whole or in part, by the Bill & Melinda Gates Foundation [OPP 1131320]. Under the grant conditions of the Foundation, a Creative Commons Attribution 4.0 Generic License has already been assigned to the Author Accepted Manuscript version that might arise from this submission.

## Conflict of Interest

The authors declare that the research was conducted in the absence of any commercial or financial relationships that could be construed as a potential conflict of interest.

## Publisher’s Note

All claims expressed in this article are solely those of the authors and do not necessarily represent those of their affiliated organizations, or those of the publisher, the editors and the reviewers. Any product that may be evaluated in this article, or claim that may be made by its manufacturer, is not guaranteed or endorsed by the publisher.
