# Supplementary figures and images for: Toll-Like Receptor-Induced Immune Responses During Early Childhood and Their Associations With Clinical Outcomes Following Acute Illness Among Infants in Sub-Saharan Africa
Source: Front Immunol. 2022 Feb 3;12:748996. doi: 10.3389/fimmu.2021.748996 (PMC8850627; doi:10.3389/fimmu.2021.748996)

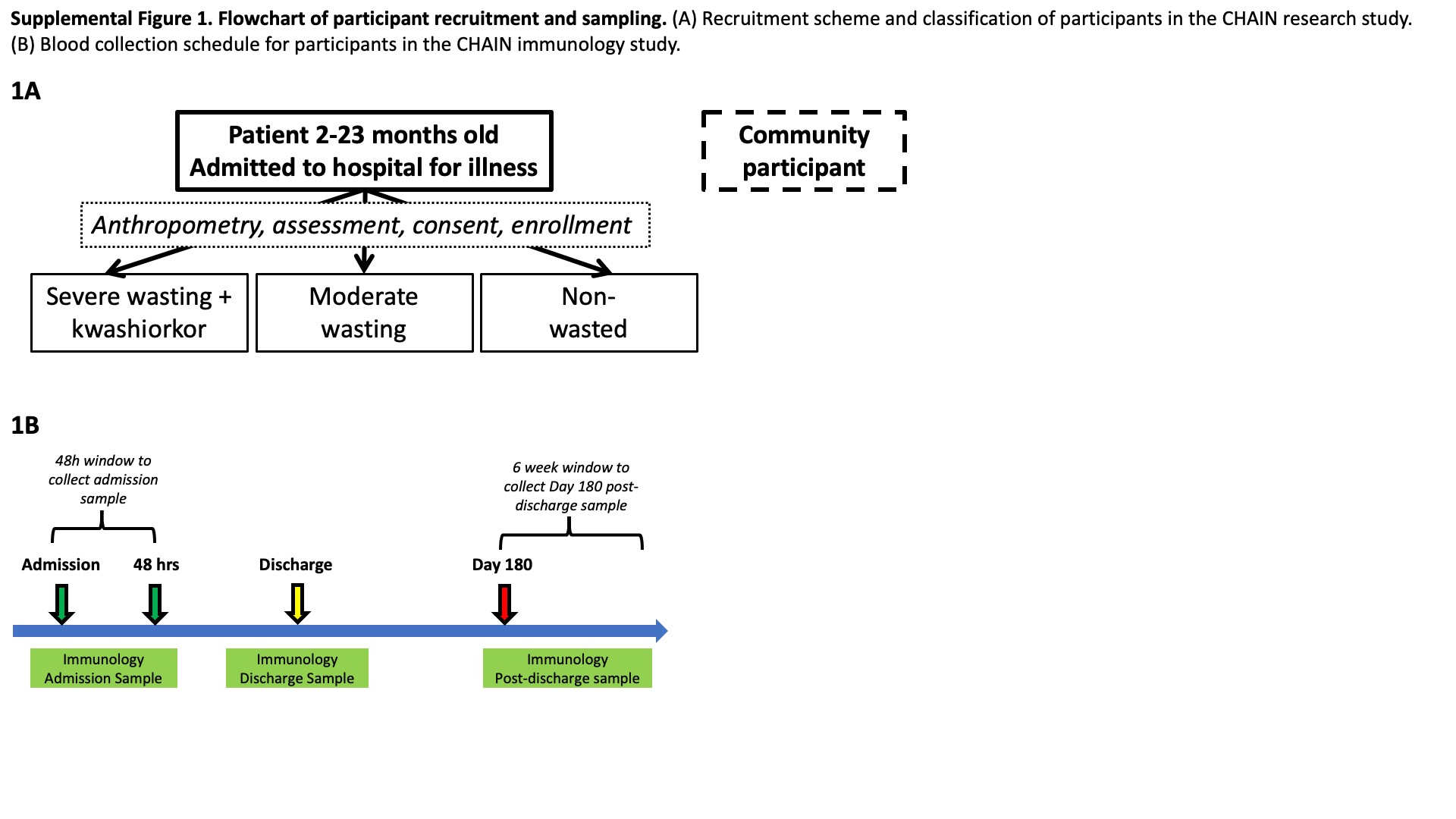

Supplement: Supplementary file 1 [file Image_1.jpeg]
